# Chronic Kidney Disease is Associated With Attenuated Plasma Metabolome Response to Oral Glucose Tolerance Testing

**DOI:** 10.1053/j.jrn.2022.09.013

**Published:** 2022-10-19

**Authors:** Armin Ahmadi, M. Nazmul Huda, Brian J. Bennett, Jorge Gamboa, Leila R. Zelnick, Lucas R. Smith, Maria Chondronikola, Daniel Raftery, Ian H. de Boer, Baback Roshanravan

**Affiliations:** * Department of Medicine, Division of Nephrology, University of California Davis, Davis, California.; † Obesity and Metabolism Research Unit, Western Human Nutrition Research Center, USDA, ARS, Davis, California.; ‡ Department of Nutrition, University of California Davis, Davis, California.; § Division of Clinical Pharmacology, Department of Medicine, School of Medicine, Vanderbilt University, Nashville, Tennessee.; ¶ Division of Nephrology and Kidney Research Institute, University of Washington, Seattle, Washington.; ** Department of Physical Medicine and Rehabilitation, School of Medicine, UCD, Davis, California.; †† Anesthesiology and Pain Medicine, University of Washington, Seattle, Washington.; ‡‡ Puget Sound Health Care System, Seattle, Washington.

**Keywords:** Chronic kidney disease, metabolism, insulin resistance

## Abstract

**Objective:**

Chronic kidney disease (CKD) is associated with decreased anabolic response to insulin contributing to protein-energy wasting. Targeted metabolic profiling of oral glucose tolerance testing (OGTT) may help identify metabolic pathways contributing to disruptions to insulin response in CKD.

**Methods:**

Using targeted metabolic profiling, we studied the plasma metabolome response in 41 moderate-to-severe nondiabetic CKD patients and 20 healthy controls at fasting and 2 hours after an oral glucose load. We used linear mixed modeling with random intercepts, adjusting for age, gender, race/ethnicity, body weight, and batch to assess heterogeneity in response to OGTT by CKD status.

**Results:**

Mean estimated glomerular filtration rate among CKD participants was 38.9 ± 12.7 mL/min per 1.73 m^2^ compared to 87.2 ± 17.7 mL/min per 1.73 m^2^ among controls. Glucose ingestion induced an anabolic response resulting in increased glycolysis products and a reduction in a wide range of metabolites including amino acids, tricarboxylic acid cycle intermediates, and purine nucleotides compared to fasting. Participants with CKD demonstrated a blunted anabolic response to OGTT evidenced by significant changes in 13 metabolites compared to controls. The attenuated metabolome response predominant involved mitochondrial energy metabolism, vitamin B family, and purine nucleotides. Compared to controls, CKD participants had elevated lactate:pyruvate (L:P) ratio and decreased guanosine diphosphate:guanosine triphosphate ratio during OGTT.

**Conclusion:**

Metabolic profiling of OGTT response suggests a broad disruption of mitochondrial energy metabolism in CKD patients. These findings motivate further investigation into the impact of insulin sensitizers and mitochondrial targeted therapeutics on energy metabolism in patients with nondiabetic CKD.

## Introduction

Chronic kidney disease (CKD) is a public health burden worldwide with the estimated global prevalence of 14% affecting more than 650 million people globally.^1^ Insulin resistance (IR) is one of the very early metabolic alterations in CKD increasing with severity of kidney disease.^2^ IR is considered a cardio-metabolic risk factor correlated with increased systemic inflammation^3^ and oxidative stress.^4^ Multiple pathophysiologic features of CKD contribute to IR in CKD including chronic inflammation,^5^ altered gut microbiome,^6^ oxidative stress,^7^ metabolic acidosis,^8^ and accumulation of uremic toxins.^6^ However, evidence for the biologic basis for IR in humans with CKD is lacking.

Although tissue insensitivity to insulin is the main contributor to IR, changes in insulin secretion and degradation have also been established in CKD.^9^ In particular, a recent study using the gold standard high-dose hyperinsulinemic-euglycemic clamp to investigate peripheral insulin sensitivity showed disturbances in insulin clearance as a principle characteristic distinguishing patients with nondiabetic CKD to controls.^10^ This study demonstrated profound alterations in the plasma metabolome response to insulin in patients with CKD compared to controls. However, understanding of the biologic basis for IR in humans with CKD at more physiologic, endogenous levels of insulin such as that elicited by oral glucose tolerance testing (OGTT) remains unknown.

OGTT provides estimates of IR^11^ but also captures additional physiologic processes by stimulating gut-derived incretin hormones known to augment insulin secretion.^12,13^ Metabolomic profiling of the response to OGTT may reveal specific metabolites and metabolic pathways underlying impaired glucose homeostasis in CKD, thus identifying therapeutic targets for improving insulin sensitivity in this vulnerable population. We performed targeted plasma metabolic profiling comparing nondiabetic patients with moderate-to-severe CKD to healthy controls during an oral glucose challenge. We hypothesize that CKD in the absence of diabetes is associated with impaired plasma metabolic response to oral glucose challenge compared to controls. Our goal was to identify specific metabolic alterations in nondiabetic CKD to better understand the metabolic and biological basis for glucose intolerance and IR in CKD.

## Materials and Methods

### Study Population and Study Design

The Study of Glucose and Insulin in Renal Disease is a cross-sectional study of glucose and insulin metabolism in moderate-to-severe nondiabetic CKD. Among the 98 recruited participants, 95 had adequate plasma samples collected for metabolomics, and of these, 61 had plasma samples before and after OGTT.^10^ Sixty one participants were included from the Study of Glucose and Insulin in Renal Disease study where 41 of them were moderate-to-severe nondiabetic CKD patients (estimated glomerular filtration rate [eGFR] < 60 mL/min per 1.73 m^2^) and 20 healthy controls (eGFR > 60 mL/min per 1.73 m^2^). Plasma metabolites were measured after overnight fasting, 2 hours after a 75g oral glucose load for OGTT and during the clamp as reported previously.^14^ Plasma biomarkers of kidney function and inflammation were measured in the fasting blood. A detailed description of the study design and distribution of the clinical phenotypes between CKD patients and healthy controls has been reported earlier.^10^

### Metabolic Profiling

Targeted metabolomics based in a liquid chromatography-tandem mass spectroscopy (LC-MS/MS) platform was performed at the Northwest Metabolomics Research Center as described previously.^15^ Briefly, blood samples were prepared by protein precipitation (methanol), centrifugation (21,694 g for 10 minutes at 4°C), drying, and reconstitution. The LC-MS/MS analyses were performed using an Agilent 1260 LC (Agilent Technologies) with a SeQuant ZIC-cHILIC column (150 × 2.1 mm × particle size 3.0 *μ*m, Merck KGaA), which was coupled to an AB Sciex QTrap 5500 MS (AB Sciex) system equipped with a standard electro-spray ionization source. Each sample was injected 2 times for analysis in positive and negative ionization modes (2 *μ*L and 10 *μ*L respectively). The flow rate was at 0.3 mL/min. Metabolites identities were determined by spiking pooled serum samples with mixtures of standard compounds. Pooled serum samples were used as quality control samples and were run after every 10 biological samples to monitor for instrument drift and signal normalization if needed. A total of 4 isotope-labeled internal standards were used to monitor sample preparation. A total of 88 metabolites were detected pre-OGTT and post-OGTT. More details for sample preparation, sample collection times, and reagents have been described earlier.^14^

### Statistical Analysis

Plasma metabolite data were available from 61 subjects at fasting and 2 hours postoral glucose load. Raw metabolite data were converted to ratio of 2 hours postoral glucose load and corresponding fasting values and checked for batch effect of the ratio values. All clinical and metabolite data were checked for normality. To account for drift of sample preparation batches, raw metabolite data were normalized using Systematic Error Removal Using Random Forest (SERRF).^16^ To account for the correlation of measurements within participants, we examined the fold changes associated with the OGTT procedure via a linear mixed model with random intercepts, regressing the log-transformed SERRF-normalized metabolite on the sample type (during OGTT vs. fasting sample), adjusting for age, gender, race/ethnicity, body weight, and batch. To evaluate whether the effect of the oral glucose challenge procedure differed between CKD and non-CKD participants, we used linear mixed effect modeling with random intercepts where SERRF-normalized metabolites were regressed on a sample type (OGTT vs. fasting), CKD status, and their interaction, additionally adjusting for covariates listed above. Data are presented as means ± SD unless otherwise indicated. Statistical analysis was performed using R 3.6.1 for windows release.^17^ *P* values were adjusted for multiple comparisons using the “Benjamini Hochberg” approach and an adjusted *P* value < .05 was considered significant for all analyses unless stated otherwise.

Pathway-associated metabolite sets enrichment and metabolite pathway analysis were performed using MetaboAnalystR v4.0^18^ with the KEGG human metabolite database.^19^ Pathway analysis using SERRF-normalized pre-OGTT and post-OGTT metabolite level was used to evaluate metabolic changes in response to OGTT in the entire cohort. Significantly altered metabolic pathway by CKD status was determined by comparing the changes in metabolite levels calculated by subtracting log-transformed post-OGTT SERRF-normalized values to pre-OGTT time points.

Differences in insulin sensitivity (Matsuda index) between CKD and controls were evaluated by Mann-Whitney U test using GraphPad Prism 9.0 (GraphPad Software, Inc., San Diego, California). Differences in plasma metabolites including guanosine diphosphate:guanosine triphosphate (GTP:GDP) ratio and lactate:pyruvate (L:P) ratio and differences in insulin secretion between fasting and 2 hours postoral glucose load were evaluated using analysis of variance for multiple hypothesis testing (Bonferroni).

Metabolite data were checked for excessive missing values by using the R package weighted gene co-expression network analysis (WGCNA)’s^20^ “goodSample-Genes” test. The correlation between kidney biomarkers was determined by Spearman correlation. The ratio of plasma metabolites postoral glucose to preglucose challenge was used for a correlation network analysis using WGCNA R package. A soft threshold approach was used with a power of 6 (based on scales-free topology) in a WGCNA default unsigned network with dynamic tree cutting (deep split = 2) and a minimum module size = 3 as parameters for the dynamic tree cut function.^21^ The module eigengene, defined as the first principal component of a module’s metabolite concentration matrix, was used to calculate the Spearman correlation between a metabolite module and kidney biomarkers.

## Results

### Characteristics of the Study Participants

The study included 41 CKD participants with a mean eGFR of 38.9 ± 12.7 mL/min per 1.73 m^2^ and 20 control subjects with a mean eGFR of 87.2 ± 17.7 mL/min per 1.73 m^2^. The mean age was 61.2 ± 12.9 years, 44% were women, and 22% were self-reported Black (Table 1). Compared with controls, participants with CKD had higher body weight, lower fat-free mass, higher BMI, daily calorie intake, and greater plasma inflammatory markers.

### Oral Glucose Challenge was Associated with a Significant Reduction in a Wide Range of Metabolites Primarily Amino Acids, Purine Nucleotides, and Dicarboxylic Acids

After adjusting for age, gender, race/ethnicity, body weight, and LC-MS batch, 65% (58/88) of the detected plasma metabolites were significantly altered postoral glucose challenge in the overall cohort (Table 2 and Table S1). The largest reductions in individual metabolite concentrations post-OGTT were observed for linoleic acid, adenosine diphosphate (ADP), and nicotinamide with a percent change of −82%, −79%, and −63%, respectively (Table 2). Only 6% (5/88) of the detected metabolites significantly increased post-OGTT compared to fasting state. These metabolites included erythrose, glucose, and kynurenate with a percent change of +53%, +28%, and +17%, respectively (Table S2).

### Insulin Secretion and Sensitivity Measured During Oral Glucose Tolerance Testing did Not Differ Among Participants with and Without Chronic Kidney Disease

**Figure 1.**
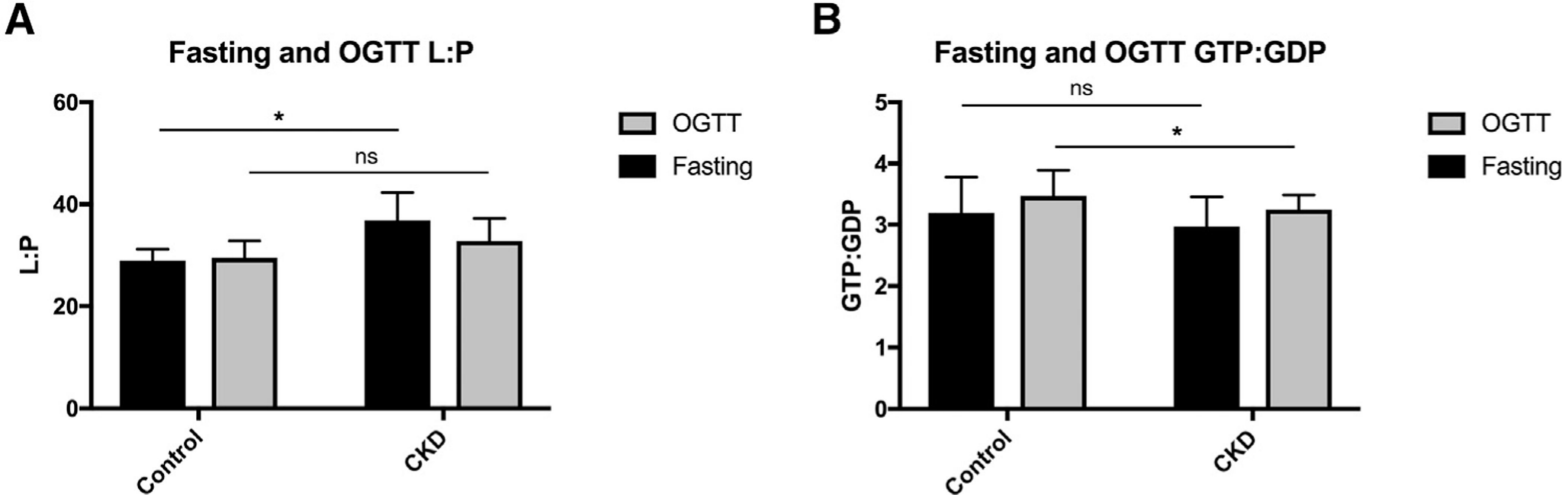
Measurements of insulin secretion and insulin sensitivity comparing CKD (n = 40) and controls (n = 20) during OGTT. (A) Plasma insulin concentration. Data points represent means and error bars represent 95% CI. (B) Matsuda index. One-way ANOVA for multiple comparison testing (A) and Mann-Whitney U test (B) were used.

We evaluated plasma insulin concentrations measured at 0, 10, 20, 30, 60,90,and120minutes after glucose ingestion and found no significant differences between CKD and controls (Fig. 1A). Similarly, the Matsuda index estimation of insulin sensitivity was not significantly different in CKD compared to controls (*P* value = .08) (Fig. 1B). The median Matsuda index in CKD and controls was 3.93 (interquartile range [IQR] of 2.44, 5.63) and 3.70 (IQR of 2.30, 9.13), respectively.

### Chronic Kidney Disease Attenuates the Plasma Metabolome Response to Glucose Challenge in Metabolites from Predominantly the Vitamin B Family, TCA Cycle Intermediates, and Purine Nucleotides

After adjustment, changes in 15% (13/88) of detected plasma metabolites were significantly altered between CKD and controls post-OGTT (Table 3 and Table S2). Overall, CKD was associated with higher plasma levels of these metabolites such as succinate, inositol, nicotinamide, and glucose in response to OGTT compared to control. A notable exception to this was kynurenate. These metabolic changes in response to OGTTare not driven by alterations in the fasting state concentrations of these metabolites in CKD versus controls (Table S3). Except for inosine monophosphate (IMP), the rest of significantly altered metabolites among CKD participants in response to OGTT were either similar or lower during fasting compared to controls (Table S3). In general, controls had on average a greater decline in plasma metabolites in response to the oral glucose challenge compared to participants with CKD. The largest difference in response to the glucose challenge comparing CKD and controls were observed in ADP, glycochenodeoxycholate, and IMP. The ratios in fold change comparing CKD to control for these metabolites were 3.7, 3.6, and 3.1, respectively (Table 3).

### The Response to the Oral Glucose Challenge Demonstrates a Broad Disruption of Amino Acid and Mitochondrial Energy Metabolism in Chronic Kidney Disease Patients

**Figure 2.**
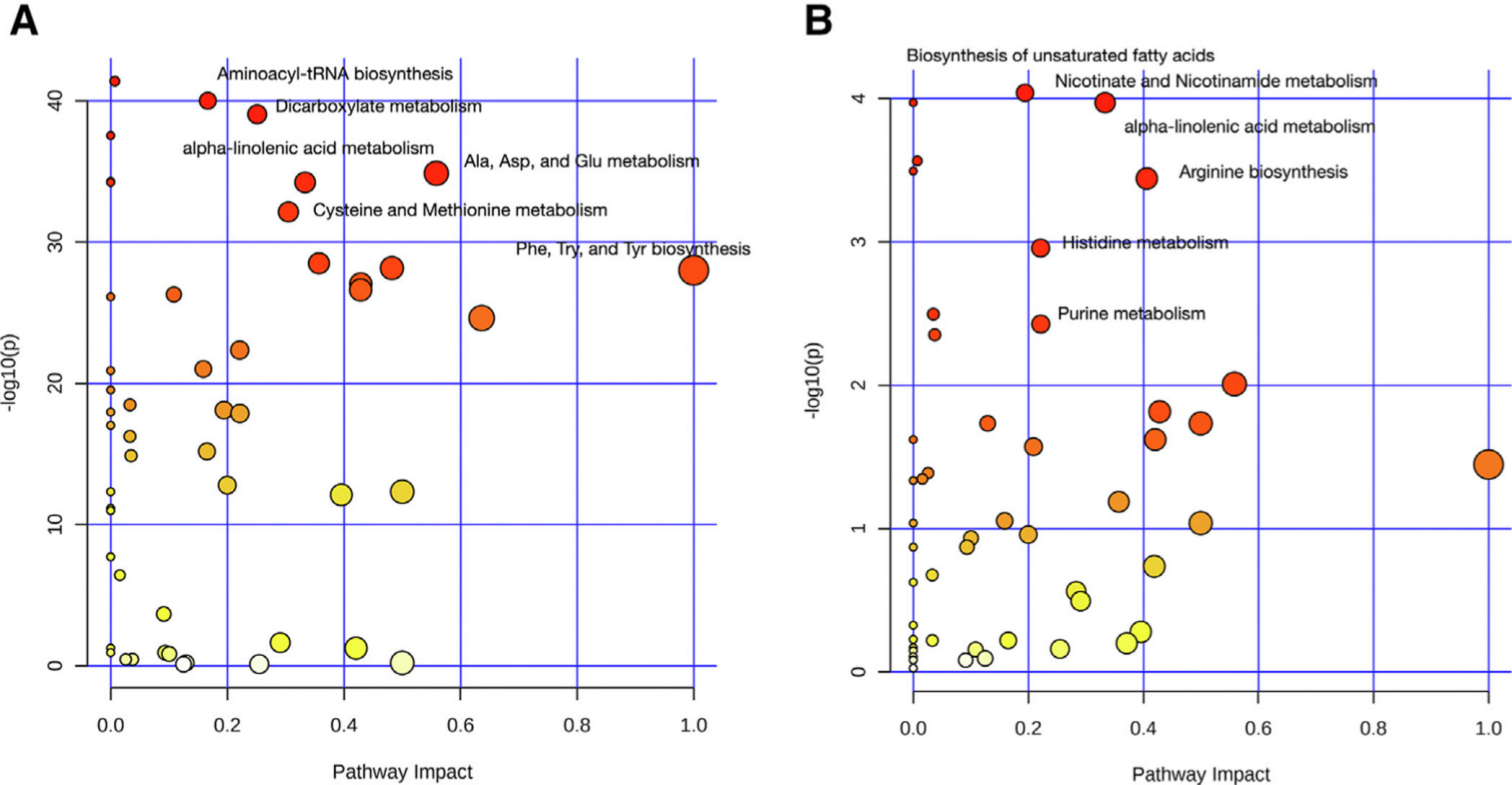
Pathway analysis of changes with oral glucose tolerance test (A) in the overall cohort (n = 61) and (B) comparing CKD (n = 41) with controls (n = 20). The size and color of the nodes represent pathway impact value and *P* value, respectively (For interpretation of the references to color in this figure legend, the reader is referred to the Web version of this article).

In the entire cohort, oral glucose challenge impacted alpha-Linoleic acid metabolism (*P* value = 3.84 × 10^−31^), aminoacyl-tRNA biosynthesis (*P* value = 1.15 × 10^−26^), and arginine biosynthesis (*P* value = 2.66 × 10^−25^) (Fig. 2A). In addition, metabolic pathways involving amino acid metabolism such as branch chain amino acids (valine, leucine, and isoleucine), phenylalanine, and histidine metabolism were also impacted. Compared to controls, participants with CKD demonstrated significant aberrations in alpha-Linoleic acid metabolism (*P* value = 2.91 × 10^−5^), nicotinamidemetabolism(*P* value = 5.18 × 10^−5^), and arginine biosynthesis (*P* value = 1.96 × 10^−4^) (Fig. 2B).

### CKD is Associated with Greater Lactate to Pyruvate (L:P) Ratio, an Indicator of Mitochondrial Respiratory Chain Impairment

An elevated lactate to pyruvate ratio suggests respiratory chain dysfunction.^22–24^ L:P ratio at fasting and post-OGTT was higher in participants with CKD compared to controls (Figure S1A). The median L:P ratio at fasting was 28.9 (IQR of 26.2, 31.2) for controls compared to 36.7 (IQR of 29.3, 42.3) among participants with CKD (*P* value = .028). The high L:P ratio was sustained post-OGTT with a median of 32.8 (IQR of 30.7, 37.2) in CKD in contrast to 29.5 (IQR of 27.8, 32.7) in controls (*P* value = .77) (Figure S1A). The difference in L:P ratio is mostly driven by an increase in pyruvate levels; however, lactate (fold change of 1.15 vs. 1.20) and pyruvate (fold change of 1.08 vs. 1.34) elevations were not meaningfully different in CKD compared to controls during OGTT (Table S2).

### CKD is Associated with Disruption of GTP:GDP Ratio Post-OGTT

The GTP:GDP ratio is known to reflect the ATP:ADP ratio.^25,26^ We looked at the GTP:GDP ratio during fasting and OGTT separately. The median GTP:GDP ratio at fasting was similar in CKD and controls (*P* value = .767) (Figure S1B). After OGTT, it is expected that the GTP and ATP levels will increase and ADP and GDP levels will decrease, thus resulting in greater GTP:GDP and ATP:ADP ratios.^25,27^ As expected, the GTP:GDP ratio increased during OGTT compared to fasting in both groups with the median of 3.11 to 3.50 in controls (*P* value = .0236) and 3.01 to 3.13 in CKD (*P* value = .207); however, the ratio did not increase meaningfully in participants with CKD compared to controls. Participants with CKD had a significantly higher post-OGTT median GTP:GDP value of 3.13 (IQR of 2.77, 3.52) compared to 3.50 (IQR of 3.11, 3.89) in controls (*P* value = .039) (Figure S1B).

### Changes in Plasma Metabolites in Response to OGTT is Associated with CKD Signature and Inflammation Markers

**Figure 3.**
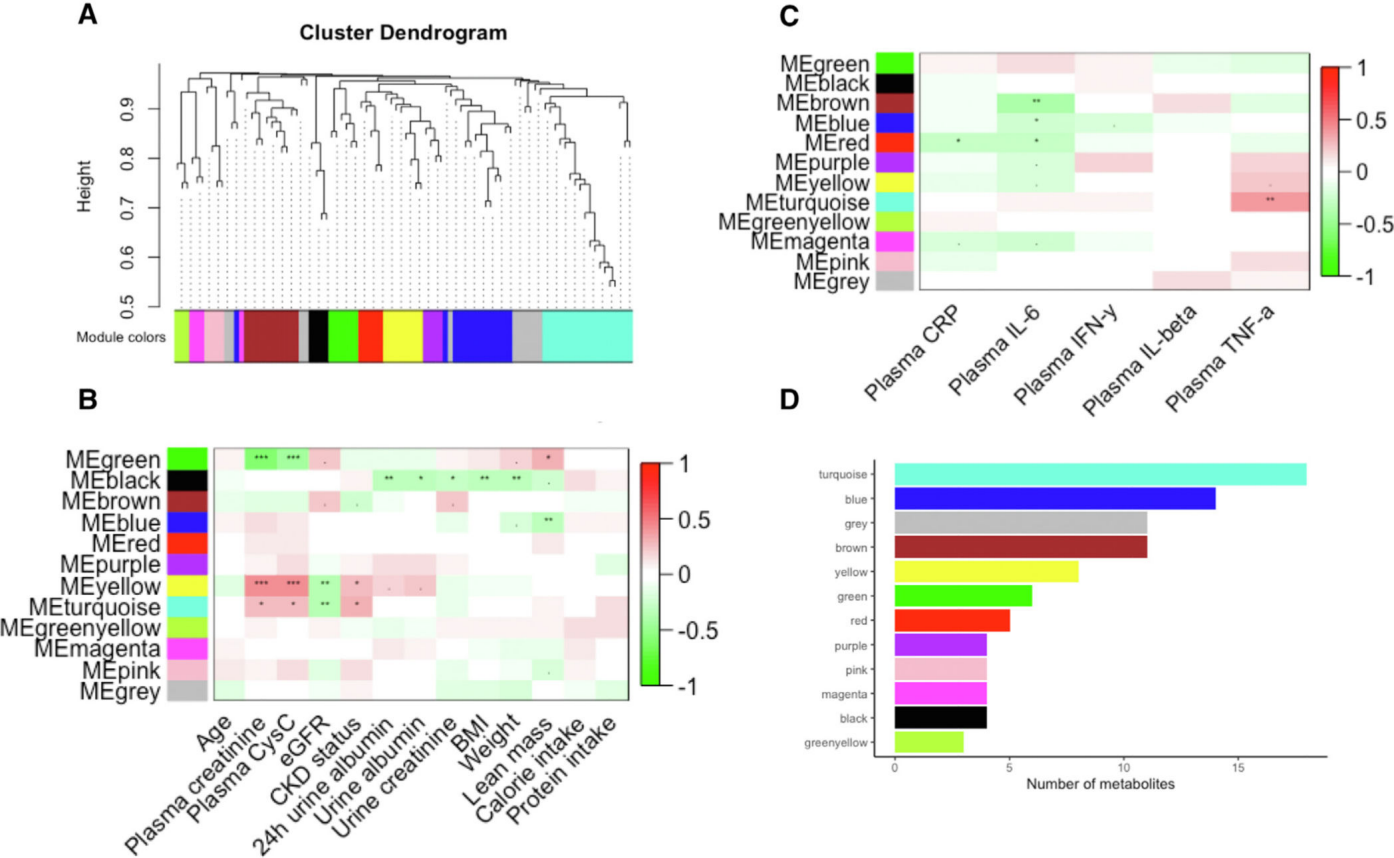
Changes in the plasma metabolic profile due to an oral glucose load is associated with kidney disease and inflammatory markers. (A) Identified metabolite modules. Correlation between plasma metabolite modules and (B) plasma biomarkers of kidney disease and (C) inflammation markers. Each row in the table corresponds to a module and each column represents a kidney biomarker, as indicated. The correlation between kidney and inflammatory biomarkers and metabolite modules were determined using spearman correlation. The correlation coefficient is color-coded as indicated in the color key legend (red = positive and green = negative correlation) “***” = *P* value < .001, “**” = *P* value < .01, “*” = *P* value < .05, “.” = *P* value < .10. (D) The number of metabolites in each of the metabolite module (For interpretation of the references to color in this figure legend, the reader is referred to the Web version of this article).

We performed WGCNA to better understand how closely related metabolite groups associate with CKD. We identified 8 metabolite modules in response to glucose load. Of the total 8 metabolite modules, those indicated in blue and green modules were significantly correlated with CKD signature and known CKD-associated inflammation markers (Fig. 3A, B, and C). Green module positively correlated with CKD status, plasma cystatin C, and plasma creatinine and negatively correlated with eGFR (Fig. 3B). Some notable metabolites from the green module include uric acid, kynurenate, and malonic acid (Table S4). The blue module also positively correlated with plasma cystatin C and plasma creatinine (Fig. 3C). Both modules also positively correlated with plasma TNF-*α* (Fig. 3C).

## Discussion

Using targeted metabolic profiling, we identified several attenuated biological and metabolic pathways in response to an oral glucose challenge in the overall cohort with evidence of marked heterogeneity by CKD status. First, we observed that compared to the fasting state OGTTresulted in a significant decrease in a wide range of metabolites including purine nucleotides and amino acids in the overall cohort. Second, patients with CKD demonstrate an attenuated plasma metabolome response to OGTT compared to controls independent of alterations in the fasting state. We observed an attenuated plasma response to OGTT particularly in vitamin B family members, mitochondrial energy metabolism, and purine metabolism. Together, our findings suggest CKD is associated with a suppressed anabolic response to glucose challenge consistent with prior findings of impaired anabolic response to insulin in this population.^14^

The application of metabolomics to the OGTT demonstrated several insights into the metabolic response to a glucose challenge. Consistent with previous studies, our cross-sectional data on 2-hour changes pre-OGTT and post-OGTT supports the known anabolic actions of insulin in promoting glycolysis and oxidative phosphorylation while suppressing proteolysis.^28,29^ In addition to tricarboxylic acid (TCA) cycle intermediates, we observed a decrease in all detected amino acids and their metabolites suggesting suppression of proteolysis and cataplerosis in the overall cohort that was comparable in magnitude to high-dose insulin clamp testing in a prior study (Table 2).

We observed significant heterogeneity by CKD status in the plasma metabolome response to glucose load revealing broad disruption in energy metabolism. In animal models, CKD leads to a postinsulin receptor defect in insulin signaling^30^ contributing to disruption of lipid,^31^ carbohydrate,^32^ and protein metabolism^33^ impacting global energy metabolism.^34^ We identified CKD-associated disruptions in GDP, ADP, and succinate; all metabolites involved in the TCA cycle. Despite equivalent levels at fasting compared to controls, these metabolites were significantly higher in CKD compared to controls post-OGTT suggesting an impaired insulin response to activate the TCA cycle (Table 3). These findings are also consistent with previous study demonstrating diminished TCA cycle activity in patients with nondiabetic CKD.^35^ Our findings underscore the importance of IR associated with metabolic defects in CKD and its potential contribution to mitochondrial dysfunction. This association is supported by a recent study suggesting that muscle-specific insulin receptor knockout in a murine model impaired mitochondrial respiration, decreased ATP production, and increased reactive oxygen species.^36^

One prominent finding suggesting mitochondrial dysfunction in CKD was an elevated L:P ratio in CKD compared to controls. The L:P ratio is known to be in near equilibrium with the NADH/NAD^+^ ratio with an elevated L:P ratio serving as an indicator for impaired mitochondrial function and TCA cycle disorder.^22,37^ An L:P ratio of > 30 has been suggestive of respiratory chain dysfunction.^24^ Increased levels of pyruvate, lactate, and L:P ratio have also been shown in patients with acute kidney failure.^38^ Glycolysis products, lactate, and pyruvate were increased in both groups in response to OGTT signaling glycolysis activation. Lactate and pyruvate however were also both more elevated in CKD compared to the controls suggesting impaired TCA cycle and oxidative phosphorylation upon glycolytic activation (Table S2). We also observed an elevated (L:P > 30) L:P ratio in CKD compared to controls both at fasting and post-OGTT suggesting mitochondrial metabolism dysfunction (Figure S1A). Taken together, our findings suggest persons with CKD have impaired mitochondrial metabolism evidenced by metabolic profiles suggesting blunted activation of TCA cycle, redox imbalance, and disruption in the mitochondrial respiratory chain.

CKD was associated with disruption in purine and pyrimidine metabolism, a crucial source of necessary energy and cofactors needed for bioenergetics and biomolecular demands of metabolism.^39^ Purines are found in biomolecules such as ATP, GTP, cyclic AMP, NADH, and coenzyme A.^40^ We noted several indicators of impairment in the purine nucleotide cycle. First, 4 metabolites involved in purine nucleotide cycle GDP, ADP, adenylosuccinate, and IMP all had an attenuated reduction in response to the glucose challenge in CKD compared to controls (Table 3). Fluctuations in ATP, ADP, and AMP and corresponding changes in GTPand GDPare regulated by purine nucleotide cycle, particularly in skeletal muscle.^41,42^ The purine cycle also enhances glycolysis and the TCA cycle via enhancing the rate of glycolysis by activating phosphofructokinase and production of fumarate.^43,44^ Second, to broadly assess disruptions in its regulatory role of energy molecules during glycolytic changes, we investigated changes in GTP:GDP ratios at fasting and during OGTT in both groups. Despite similar fasting GTP:GDP ratios in CKD compared to controls, CKD patients had lower GTP:GDP ratio post-OGTT suggesting an impairment in the purine nucleotide cycle interfering with energy generation. These findings in the plasma are hypothesis generating and motivate interrogation in human tissues to confirm a mechanistic link between CKD and impaired energy metabolism specifically focusing on the impact of CKD on ATP:ADP ratio and purine and pyrimidine metabolism.

CKD was also associated with disruption of metabolites in the vitamin B family with OGTT. Vitamin B family members are essential for metabolism involved in major metabolic pathways acting as cofactors in catabolic and anabolic pathways including carbohydrate, protein, and fat metabolism.^45^ We found a decrease in vitamin B family derivatives in response to OGTT in both groups. Among vitamin B family members, the most significant decrease post-OGTT was observed in nicotinamide (a form of vitamin B3), important in various oxidation/reduction reactions. Compared to controls where nicotinamide levels profoundly decreased in response to OGTT, the levels of nicotinamide in participants with CKD remained elevated (Table 3) consistent with our prior findings using insulin clamp testing.^14^ Our data confirm findings from these other studies showing nicotinamide and other NAD ^+^ catabolites accumulating in uremic patients suggesting an insulin-mediated defect to use nicotinamide during anabolic reactions. Dynamic changes in other B vitamins were also detected post-OGTT (Table S2). Together, the attenuated response of vitamin B family members post-OGTT in CKD versus controls points toward impairment in activating insulin-mediated anabolic pathways involving accumulation of these cofactors in patients with CKD.

There were several metabolic profile changes in response to the glucose challenge characteristic of CKD^46^ and levels of CKD-associated inflammation markers identified using WGCNA analysis.^47^ A module consisting of malonate, leucic acid, IMP, kynurenate, uric acid, and inositol had a significant positive correlation with plasma creatinine, plasma cystatin C, CKD status, and a significant negative correlation with eGFR (Fig. 3B). This module also had a significant positive correlation with TNF-*α* levels (Fig. 3C). Interestingly, uremic retention solutes and uremic toxins including inositol, malonate, and uric acid make up a considerable number of correlating metabolites in this module. Inositol is involved in energy metabolism and considered a uremic retention solute. It was attenuated post-OGTT in the CKD group compared to controls and part of the green module correlating with CKD status and TNF-*α* levels. Serum inositol levels have been negatively correlated with GFR^48,49^ and implicated to have an adverse impact on renal progression particularly among patients with type 2 diabetes where higher myo-inositol levels associates with greater likelihood of progression to end-stage renal disease.^50^ In summary, WGCNA analysis suggests uremic toxins are strongly associated with disruption in response to glucose challenge in CKD compared to controls and linked to inflammatory markers in our cohort.

Our study has several notable strengths and limitations. First, we assessed dynamic changes in response to glucose challenge and accounted for several potential confounding factors by adjusting for age, gender, race, body weight, and batch in our analysis. Second, we applied targeted metabolic profiling to assess a broad range of attenuated metabolic pathways impacted by physiological effects of insulin and identified its potential disturbed mechanisms in CKD. Our study also had some notable limitations. First, we did not investigate changes in human tissue relying instead on changes in the plasma metabolome. Changes in the plasma metabolome may not correlate with tissue or organ-specific metabolic alterations. Second, plasma ATP measurements were not available during fasting and OGTT. We relied on GTP:GDP ratios as a reflection of the changes in ATP:ADP. Third, we cannot rule out the potential impact of residual confounding by differences in unmeasured characteristics between CKD and controls. Finally, we are unable to precisely identify the potential differences in the incretin-induced secretion of insulin between our two groups. Further studies are necessary to interrogate the association of reduced activation of TCA cycle, redox imbalance, and electron transport chain efficiency in human tissues especially in the skeletal muscle.

In summary, numerous plasma metabolites are altered in response to oral glucose challenge in CKD. The response to glucose challenge in CKD is associated with disruption in plasma levels of TCA cycle intermediates, purine nucleotide cycle metabolites, and vitamin B family members relative to controls. These alterations highlight a remarkable overlap in the metabolic pathways, the magnitude, and the pattern of changes in plasma metabolites compared to our hyperinsulinemic-euglycemic clamp study among CKD. Both procedures resulted in similar changes in vitamin B family including nicotinamide and biotin. In addition, impairment in taurine metabolism was also replicated during glucose challenge. Overall, both studies highlight impairments in TCA cycle intermediates, amino acid metabolism, purine metabolism, and vitamin B family as the consequence of blunted insulin anabolic response in CKD. Together, our findings from response to OGTT and insulin clamp point to abnormal mitochondrial energy metabolism as the main mechanism of the impaired anabolic response to insulin in CKD. This abnormal metabolic profile in response to glucose challenge adds to prior studies indicating the IR in CKD is predominantly characterized by a depressed anabolic response and disruption in energy metabolism. Given recent evidence for clinical benefits of insulin sensitizers, there is an urgent need for future studies interrogating the pleotropic effects of insulin sensitizers on mitochondrial energy metabolism and anabolism in CKD.

### Practical Application

The current investigation applies plasma metabolomics to describing the metabolic changes in response to a standard oral glucose tolerance testing comparing persons with nondiabetic CKD to controls. Our findings suggest that the response to oral glucose challenge in CKD may be associated with disruption in both protein anabolic response to insulin and mitochondrial energy metabolism. Uremic toxins may link inflammation with the disruption of mitochondrial energy metabolism and the attenuated anabolic response to insulin in CKD. These findings add an insight into potential mechanisms underlying IR and protein energy wasting in persons living with nondiabetic CKD.

## Supplementary Material

supplementary material

## Figures and Tables

**Table 1.** Demographic Characteristics by Chronic Kidney Disease Status of Analytic Population (n = 61)

	Controls	Chronic Kidney Disease
Characteristic	N = 20	N = 41
Demographics Age (yr), mean (SD)	60.3 (12.9)	61.6 (14.3)
Female, number (%)	9 (45)	18 (44)
Race/ethnicity, number (%) White	16 (80)	28 (68)
Black	4 (20)	10 (24)
Asian/Pacific Islander	0 (0)	3 (7)
Physical characteristics, mean (SD) Weight (Kg)	85.9 (21.4)	89.5 (20.3)
Fat-free mass (kg)	54.3 (11.9)	51.9 (11.5)
BMI, mean (SD)	28.7 (6.43)	30.2 (6.16)
Dietary data Total calorie intake (kcal), mean (SD)	1987.1 (615.3)	1819.0 (573.1)
Daily protein intake (g), mean (SD)	75.0 (23.1)	74.4 (29.8)
Laboratory data eGFR (mL/min/1.73m2), mean (SD)	87.2 (17.7)	38.9 (12.7)
Serum creatinine (mg/dL), median (IQR)	0.9 (0.81–1.0)	1.67 (1.50–1.80)
Serum cystatin C (mh/dL), median (IQR)	0.89 (0.68–0.97)	1.49 (1.40–1.81)
Urine albumin excretion rate (mg/24 hours), median (IQR)	5 (3.4–7.8)	127.15 (20.57–422.85)
CRP (mg/dL), median (IQR)	0.11 (0.05–0.25)	0.23 (0.13–0.27)
IL-1*β* (pg/mL), median (IQR)	0.09 (0.05–0.14)	0.12 (0.08–0.15)
IL-6 (pg/mL), median (IQR)	0.84 (0.56–1.19)	0.99 (0.83–1.51)
IFN-ɣ (pg/mL), median (IQR)	2.24 (1.09–4.15)	3.26 (2.59–5.62)
TNF-*α* (pg/mL), median (IQR)	1.49 (0.75–1.95)	2.53 (2.23–2.85)
Baseline medication Antihypertensive medication, number (%)	8 (40.0)	31 (86.1)
RAASi	4 (20)	22 (61.11)
Diuretic	2 (10)	15 (41.6)
Statin	3 (15)	14 (38.9)
Beta blocker	2 (10)	12 (33.3)
CCBs	2 (10)	18 (50)

CCB, calcium channel blocker; CRP, C-reactive protein; eGFR, estimated glomerular filtration rate; IQR, interquartile range; RAASi, renin-angiotensin-aldosterone system inhibitor; SD, standard deviation.

Chronic kidney disease was defined as estimated glomerular filtration rate < 60 mL/min per 1.73 m^2^; controls as ≥ 60 mL/min per 1.73 m^2^.

**Table 2.** Differences in Plasma Metabolites During OGTT, Compared With the Fasting State

Metabolite	Fold Change Between the OGTT/Fasting States (95% CI)	*P* Value
Linolenic acid	0.18 (0.15, 0.21)	<.001
ADP	0.21 (0.14, 0.32)	<.001
Niacinamide	0.37 (0.29, 0.48)	<.001
Malonic acid	0.38 (0.28, 0.51)	<.001
Taurine	0.49 (0.41, 0.58)	<.001
Aspartic acid	0.51 (0.43, 0.6)	<.001
Adenylosuccinate	0.53 (0.44, 0.65)	<.001
iso-Leucine	0.55 (0.5, 0.6)	<.001
Leucine	0.56 (0.51, 0.61)	<.001
Citrulline	0.58 (0.53, 0.62)	<.001
PGE	0.6 (0.53, 0.69)	<.001
Succinate	0.61 (0.55, 0.68)	<.001
Tyrosine	0.65 (0.61, 0.69)	<.001
Sorbitol	0.66 (0.6, 0.72)	<.001
Methionine	0.67 (0.62, 0.71)	<.001
Cystathionine	0.7 (0.59, 0.84)	<.001
Glutamic acid	0.7 (0.6, 0.82)	<.001
2-Hydroxyglutarate	0.7 (0.62, 0.78)	<.001
Serine	0.71 (0.66, 0.76)	<.001
MethylSuccinate	0.72 (0.67, 0.78)	<.001

Top 20 based on *P* value and fold change are shown in the table. Fold changes are adjusted to age, gender, race, body weight, and batch effect. The fold changes are compared to the fasting state. For example, Nicotinamide has a fold change of 0.37 meaning it had a 63% reduction post OGTT compared to the fasting state.

**Table 3.** Differences in Plasma Metabolites Response Post Oral Glucose Tolerance Test by Chronic Kidney Disease Status

Metabolite	Fold change between OGTT/fasting (95% CI) in Control	Fold Change Between OGTT/Fasting (95% CI) in CKD	*P* Value For Interaction	Pathway
Succinate	0.48 (0.37, 0.62)	0.78 (0.71, 0.85)	<.001	TCA Cycle
Taurine	0.41 (0.28, 0.6)	0.75 (0.66, 0.85)	.0032	Amino acids metabolism/Sulfur metabolism
Adenylosuccinate	0.41 (0.26, 0.65)	0.84 (0.72, 0.98)	.0037	Nucleotide/Purine metabolism
ADP	0.17 (0.07, 0.42)	0.63 (0.47, 0.86)	.0063	Nucleotide/Purine metabolism
Biotin	0.74 (0.59, 0.93)	1.02 (0.94, 1.1)	.011	Vitamins
Niacinamide	0.35 (0.2, 0.6)	0.7 (0.58, 0.84)	.019	Vitamins
Glycochenodeoxycholate	0.47 (0.16, 1.35)	1.69 (1.18, 2.42)	.025	Bile acid metabolism
Inositol	0.92 (0.85, 0.99)	1 (0.98, 1.03)	.026	Glucose/inositol metabolism
Kynurenate	1.07 (0.82, 1.4)	0.78 (0.71, 0.85)	.026	Amino Acid metabolism/Try
IMP	0.18 (0.07, 0.48)	0.55 (0.39, 0.76)	.037	Nucleotide/Purine metabolism
Uridine	0.69 (0.49, 0.96)	0.99 (0.89, 1.11)	.043	Nucleotide/Pyrimidine metabolism
Glucose	1.07 (0.88, 1.31)	1.34 (1.25, 1.43)	.043	Glycolysis/sugar
GDP	0.73 (0.59, 0.89)	0.9 (0.84, 0.97)	.05	Nucleotide/Purine metabolism

Fold changes between OGTT and fasting states by CKD status are adjusted for age, gender, race, weight, and batch. Fold changes are compared to the fasting state. For example, a fold change of 0.78 indicates a 22% reduction in metabolite level. *P* value for interaction tests the heterogeneity in fold change by CKD status.

## References

[1] Bikbov B, Purcell CA, Levey AS, Global, regional, and national burden of chronic kidney disease, 1990–2017: a systematic analysis for the Global Burden of Disease Study 2017. The Lancet. 2020;395:709–733. 10.1016/S0140-6736(20)30045-3 PMC7049905 32061315

[2] Spoto B, Pisano A, Zoccali C. Insulin resistance in chronic kidney disease: a systematic review. Am J Physiol Ren Physiol. 2016;311:F1087–F1108. Accessed October 22, 2016. 10.1152/ajprenal.00340.2016 27707707

[3] de Luca C, Olefsky JM. Inflammation and insulin resistance. FEBS Lett. 2008;582:97–105. PubMed Accessed November 29, 2007. 18053812 10.1016/j.febslet.2007.11.057 PMC2246086

[4] Hurrle S, Hsu WH. The etiology of oxidative stress in insulin resistance. Biomed J. 2017;40:257–262. PubMed Accessed November 8, 2017. 29179880 10.1016/j.bj.2017.06.007 PMC6138814

[5] Senn JJ, Klover PJ, Nowak IA, Mooney RA. Interleukin-6 induces cellular insulin resistance in hepatocytes. Diabetes. 2002;51:3391–3399. PubMed Accessed November 28, 2002. 12453891 10.2337/diabetes.51.12.3391

[6] Koppe L, Pillon NJ, Vella RE, p-Cresyl sulfate promotes insulin resistance associated with CKD. J Am Soc Nephrol. 2013;24:88–99. PubMed Accessed January 1, 2013. 23274953 10.1681/ASN.2012050503 PMC3537215

[7] Wright VP, Reiser PJ, Clanton TL. Redox modulation of global phosphatase activity and protein phosphorylation in intact skeletal muscle. J Physiol. 2009;587:5767–5781. PubMed Accessed October 21, 2009. 19841000 10.1113/jphysiol.2009.178285 PMC2805384

[8] Walker BG, Phear DN, Martin FI, Baird CW. Inhibition OF insulin BY acidosis. Lancet. 1963;2:964–965. PubMed Accessed November 9, 1963. 14059049 10.1016/s0140-6736(63)90670-6

[9] Rahhal M-N, Gharaibeh NE, Rahimi L, Ismail-Beigi F. Disturbances in insulin–glucose metabolism in patients with advanced renal disease with and without diabetes. The J Clin Endocrinol Metab. 2019;104:4949–4966. 31162534 10.1210/jc.2019-00286

[10] De Boer IH, Zelnick L, Afkarian M, Impaired glucose and insulin homeostasis in moderate-severe CKD. J Am Soc Nephrol. 2016;27:2861–2871. 26823551 10.1681/ASN.2015070756 PMC5004653

[11] Reaven GM, Brand RJ, Chen YD, Mathur AK, Goldfine I. Insulin resistance and insulin secretion are determinants of oral glucose tolerance in normal individuals. Diabetes. 1993;42:1324–1332. PubMed Accessed September 1, 1993. 8349044 10.2337/diab.42.9.1324

[12] Perley MJ, Kipnis DM. Plasma insulin responses to oral and intravenous glucose: studies in normal and diabetic sujbjects. J Clin Invest. 1967;46:1954–1962. PubMed Accessed December 1, 1967. 6074000 10.1172/JCI105685 PMC292948

[13] McIntyre N, Holdsworth CD, Turner DS. New interpretation of oral glucose tolerance. Lancet. 1964;2:20–21. PubMed Accessed July 4, 1964. 10.1016/s0140-6736(64)90011-x 14149200

[14] Roshanravan B, Zelnick LR, Djucovic D, Chronic kidney disease attenuates the plasma metabolome response to insulin. JCI Insight. 2018;3:e122219. PubMed Accessed August 24, 2018. 10.1172/jci.insight.122219 PMC6141172 30135309

[15] Zhu J, Djukovic D, Deng L, Colorectal cancer detection using targeted serum metabolic profiling. J Proteome Res. 2014;13:4120–4130. PubMed Accessed August 16, 2014. 25126899 10.1021/pr500494u

[16] Fan S, Kind T, Cajka T, Systematic error Removal using random forest for normalizing large-scale untargeted lipidomics data. Anal Chem. 2019;91:3590–3596. PubMed Accessed February 14, 2019. 30758187 10.1021/acs.analchem.8b05592 PMC9652764

[17] R Core T. R: A Language and Environment for Statistical Computing. Austria, 2019: R Foundation for Statistical Computing. http://www.R-project.org. Accessed November 14, 2022.

[18] Chong J, Soufan O, Li C, MetaboAnalyst 4.0: towards more transparent and integrative metabolomics analysis. Nucleic Acids Res. 2018;46:W486–W494. PubMed Accessed May 16, 2018. 29762782 10.1093/nar/gky310 PMC6030889

[19] Ogata H, Goto S, Fujibuchi W, Kanehisa M. Computation with the KEGG pathway database. Biosystems. 1998;47:119–128. 9715755 10.1016/s0303-2647(98)00017-3

[20] Langfelder P, Horvath S. WGCNA: an R package for weighted correlation network analysis. BMC Bioinformatics. 2008;9:559. 19114008 10.1186/1471-2105-9-559 PMC2631488

[21] Langfelder P, Zhang B, Horvath S. Defining clusters from a hierarchical cluster tree: the dynamic tree cut package for R. Bioinformatics. 2007;24:719–720. 18024473 10.1093/bioinformatics/btm563

[22] Hancock MR. Mitochondrial dysfunction and the role of the non-specialist laboratory. Ann Clin Biochem. 2002;39:456–463. PubMed 12227851 10.1258/000456302320314467

[23] Gropman AL. Diagnosis and treatment of childhood mitochondrial diseases. Curr Neurol Neurosci Rep. 2001;1:185–194. 11898515 10.1007/s11910-001-0015-9

[24] Neurosurgery O. Lactate to pyruvate ratio 2018. https://operativeneurosurgery.com/doku.php?id5lactate_to_pyruvate_ratio. Accessed November 1, 2022.

[25] Detimary P, Van den Berghe G, Henquin JC. Concentration dependence and time course of the effects of glucose on adenine and guanine nucleotides in mouse pancreatic islets. J Biol Chem. 1996;271:20559–20565. PubMed Accessed August 23, 1996. 8702800 10.1074/jbc.271.34.20559

[26] Kibbey RG, Pongratz RL, Romanelli AJ, Wollheim CB, Cline GW, Shulman GI. Mitochondrial GTP Regulates glucose-Stimulated insulin secretion. Cell Metab. 2007;5:253–264. 17403370 10.1016/j.cmet.2007.02.008 PMC1876711

[27] Windisch RM, Pax PR, Bracken MM. Variations in blood ATP after oral administration of glucose, in individuals diagnosed as normal, equivocal, or diabetic according to the glucose tolerance sum principle. Clin Chem. 1970;16:941–944. PubMed Accessed November 1, 1970. 5473555

[28] Wang Q, Jokelainen J, Auvinen J, Insulin resistance and systemic metabolic changes in oral glucose tolerance test in 5340 individuals: an interventional study. BMC Med. 2019;17:217. 31779625 10.1186/s12916-019-1440-4 PMC6883544

[29] Ho JE, Larson MG, Vasan RS, Metabolite profiles during oral glucose challenge. Diabetes. 2013;62:2689–2698. 23382451 10.2337/db12-0754 PMC3717862

[30] Bailey JL, Zheng B, Hu Z, Price SR, Mitch WE. Chronic kidney disease causes defects in signaling through the insulin receptor substrate/phosphatidylinositol 3-kinase/Akt pathway: implications for muscle atrophy. J Am Soc Nephrol. 2006;17:1388–1394. PubMed Accessed April 14, 2006. 16611720 10.1681/ASN.2004100842

[31] Tangvarasittichai S. Oxidative stress, insulin resistance, dyslipidemia and type 2 diabetes mellitus. World J Diabetes. 2015;6:456–480. PubMed Accessed April 22, 2015. 25897356 10.4239/wjd.v6.i3.456 PMC4398902

[32] Ikee R, Hamasaki Y, Oka M, Glucose metabolism, insulin resistance, and renal pathology in non-diabetic chronic kidney disease. Nephron Clin Pract. 2008;108:c163–c168. PubMed Accessed February 9, 2008. 18259103 10.1159/000115329

[33] Siew ED, Ikizler TA. Determinants of insulin resistance and its effects on protein metabolism in patients with advanced chronic kidney disease. Contrib Nephrol.2008;161:138–144. Accessed May 3, 2008. 18451670 10.1159/000130659

[34] Thomas SS, Zhang L, Mitch WE. Molecular mechanisms of insulin resistance in chronic kidney disease. Kidney Int. 2015;88:1233–1239. PubMed Accessed October 8, 2015. 26444029 10.1038/ki.2015.305 PMC4675674

[35] Hallan S, Afkarian M, Zelnick LR, Metabolomics and gene expression analysis reveal Down-regulation of the Citric acid (TCA) cycle in non-diabetic CKD patients. EBioMedicine. 2017;26:68–77. PubMed Accessed November 13, 2017. 29128444 10.1016/j.ebiom.2017.10.027 PMC5832558

[36] Bhardwaj G, Penniman CM, Jena J, Insulin and IGF-1 receptors regulate complex I-dependent mitochondrial bioenergetics and supercomplexes via FoxOs in muscle. J Clin Invest. 2021;131:e146415. PubMed Accessed August 4, 2021. 10.1172/JCI146415 PMC8439595 34343133

[37] Munnich A, Rötig A, Chretien D, Saudubray JM, Cormier V, Rustin P. Clinical presentations and laboratory investigations in respiratory chain deficiency. Eur J Pediatr. 1996;155:262–274. PubMed Accessed April 1, 1996. 8777918 10.1007/BF02002711

[38] Ando M, Shimizu K. [Acute renal failure with lactic acidosis]. Nihon Jinzo Gakkai Shi. 1990;32:729–737. PubMed Accessed June 1, 1990. 2214321

[39] Pedley AM, Benkovic SJ. A New View into the regulation of purine metabolism: the Purinosome. Trends Biochem Sci. 2017;42:141–154. 28029518 10.1016/j.tibs.2016.09.009 PMC5272809

[40] Kumari A. Chapter 17 - purine Structures. In: Kumari A, ed. Sweet Biochemistry. Academic Press; 2018:89–91.

[41] Tornheim K. Oscillations of the glycolytic pathway and the purine nucleotide cycle. J Theor Biol. 1979;79:491–541. PubMed 159983 10.1016/0022-5193(79)90240-6

[42] Tornheim K, Lowenstein JM. The purine nucleotide cycle. Control of phosphofructokinase and glycolytic oscillations in muscle extracts. J Biol Chem. 1975;250:6304–6314. PubMed Accessed August 25, 1975. 169235

[43] Arinze IJ. Facilitating understanding of the purine nucleotide cycle and the one-carbon pool: Part I: the purine nucleotide cycle. Biochem Mol Biol Education. 2005;33:165–168. 10.1002/bmb.2005.494033032469 21638570

[44] Bhagavan NV. Chapter 27 - nucleotide metabolism. In: Bhagavan NV, ed. Medical Biochemistry. Fourth Edition San Diego: Academic Press; 2002:615–644.

[45] Vitamins Important for Metabolism. https://med.libretexts.org/@go/page/2620. Accessed November 14, 2022.

[46] Lopez-Giacoman S, Madero M. Biomarkers in chronic kidney disease, from kidney function to kidney damage. World J Nephrol. 2015;4:57–73. PubMed Accessed February 11, 2015. 25664247 10.5527/wjn.v4.i1.57 PMC4317628

[47] Muslimovic A, Rasic S, Tulumovic D, Hasanspahic S, Rebic D. Inflammatory markers and Procoagulants in chronic renal disease stages 1–4. Med Arch. 2015;69:307–310. PubMed Accessed December 2, 2015. 26622082 10.5455/medarh.2015.69.307-310 PMC4639342

[48] Sekula P, Goek ON, Quaye L, A metabolome-wide association studyof kidney function and disease in the general population. J Am Soc Nephrol. 2016;27:1175–1188. PubMed Accessed October 10, 2015. 26449609 10.1681/ASN.2014111099 PMC4814172

[49] Sui W, Li L, Che W, A proton nuclear magnetic resonance-based metabonomics study of metabolic profiling in immunoglobulin a nephropathy. Clinics (Sao Paulo). 2012;67:363–373. PubMed Accessed April 24, 2012. 22522762 10.6061/clinics/2012(04)10 PMC3317244

[50] Niewczas MA, Sirich TL, Mathew AV, Uremic solutes and risk of end-stage renal disease in type 2 diabetes: metabolomic study. Kidney Int. 2014;85:1214–1224. PubMed Accessed January 17, 2014. 24429397 10.1038/ki.2013.497 PMC4072128

